# The geography of talent development

**DOI:** 10.3389/fspor.2022.1031227

**Published:** 2022-10-06

**Authors:** David J. Hancock, Matthew Vierimaa, Ashley Newman

**Affiliations:** ^1^School of Human Kinetics and Recreation, Memorial University of Newfoundland, St. John's, NL, Canada; ^2^School of Kinesiology, Acadia University, Wolfville, NS, Canada

**Keywords:** birth, birthplace, youth, community, social

## Abstract

Geography (i.e., birthplace) is one of many factors that influence talent development. When one's birthplace leads to advantages in sport participation or performance, it is called a birthplace effect. Nearly two decades of committed research has revealed that birthplace effects are pervasive across sports and countries. Recently, researchers have attempted to better understand birthplace effects by considering various metrics that serve as proxies for birth advantages; for instance, population size, population density, and proximity to sport clubs. Underlying mechanisms that explain birthplace effects include infrastructure (e.g., environment and facilities) and social structure (e.g., family and safety), though contextual differences across existing research (e.g., sports and countries) make it difficult to fully explain the effects. Herein, we provide more depth regarding these elements of birthplace effects, while also presenting new data on “talent hotspots”; that is, communities with optimal population *and* density for talent development.

## Introduction

When considering the factors that influence elite talent development in sport, most would point to genetics, intrinsic motivation, passion, and work ethic. Perhaps fewer people might identify other contributing factors such as coaching and birthdate. Often overlooked, albeit quite important, is the influence that geography (i.e., birthplace) has on talent development. A seminal study by Côté et al. (1) firmly placed birthplace effects on sport scientists' research agendas, leading to dozens of studies on the topic conducted since then. In this chapter, we will outline some of these studies to showcase what is known about birthplace effects in sport. Following, we will describe some of the variability in birthplace effects results, identify weaknesses and gaps related to birthplace effects research, and explain some recent findings on “talent hotspots.”

## The birthplace effect

The process to identify birthplace effects is relatively simple. First, a sample of athletes is selected for examination (e.g., handball players in Spain). Second, the athletes are placed into various categories, based on the population of their birthplaces (e.g., 25,000–49,999). Next, the percentage of athletes in each category is calculated (e.g., 12% of athletes in the sample were born in the 25,000–49,999 category). Following, the percentage of the general population born in each population category is calculated (e.g., 5% of the general population is born in the 25,000–49,999 category). Finally, across each population category, the distribution of athletes is compared to the distribution of the general population (e.g., 12 vs. 5% in the 25,000–49,999 category). The mathematical calculations to identify if the distribution differences are “significant” or “meaningful” require more depth of understanding, which goes beyond the necessary description for this chapter. Suffice to say, these steps allow one to conclude if athletes born in a certain population category are (1) more, (2) less, or (3) no more likely to achieve elite sport status.

Though one's birthplace is rather arbitrary, researchers have uncovered birthplace effects spanning countries and sports. Côté et al. (1) conducted one of the earliest studies on birthplace effects, exploring American athletes who reached professional leagues in ice hockey, basketball, baseball, and golf. Regardless of sport, their findings indicated that, compared to any other population category, people born in communities of 50,000–99,999 residents were much more likely to attain professional athlete status (11 times more likely for basketball and golf; 19 times more likely for ice hockey; and 21 times more likely for baseball). Meanwhile, across all sports, people born in cities of 500,000 or more residents were far less likely to attain professional athlete status. Given the findings, the authors proposed that the nature of smaller cities must offer advantages for talent development that are absent in other population categories. They further suggested that the nature of sport in large cities (i.e., stratified competitions, time commitments, expenses, etc…) actually inhibits talent development.

In the years following Côté et al.'s (1) study, there was an influx of research on birthplace effects. Mainly, sport scientists aimed to understand the pervasiveness of the effect to better catalog the role of geography in athletic talent development. Baker and Logan (2), for instance, uncovered birthplace effects for Canadian- and American-born athletes who were drafted into the National Hockey League. Other sports with noted birthplace effects based on population size include volleyball (3), soccer (4), handball (5), gridiron football (6), and cricket (7). Whereas most research focused on male athletes, the effect has been discovered among female athletes as well, specifically for athletes in soccer, golf, basketball, handball, and volleyball (4, 8). Likewise, while most researchers focus on birthplace as a conduit to elite athletic success, other studies have noted the influence of one's place of birth on sport dropout [i.e., more likely to drop out of ice hockey if born in cities with 500,000 or more residents; (9)] and participation [i.e., more likely to participate in team and individual sports if born in communities of 10,000–100,000 residents; (10)]. Strengthening the case that birthplace effects are pervasive, this effect has been found in several countries including Canada (9), United States (1), Portugal (3), Israel (8), Brazil (11), United Kingdom (7), and Germany (10), to name a few.

## Variations in birthplace effects

Whereas birthplace effects (as measured by population size) are fairly consistent, there is considerable variability in the advantaged population categories across existing studies. For instance, some studies show optimal birthplaces being communities of 50,000–99,999 residents (1), with others indicating 200,000–399,999 residents (3) or 500,000 or more residents (6) as optimal. Variations in findings are not surprising as the structure of communities varies greatly by continent (e.g., comparing North American and European community structures), and even within one continent (e.g., comparing German and Norwegian community structures). Such variations have spurred birthplace effects researchers to use different metrics to examine trends. One such method is considering population density as opposed to absolute population size, which might be more reflective of the structure of one's community. Hancock et al. (3) advocated for this approach, noting an example comparing Paris and Toronto, which share similar population sizes, but Paris has four times the population density.

Rossing et al. (12) explored birthplace effects among elite male (12 years old and younger) Danish soccer and handball players, using population density as a metric. Therein, the authors reported that soccer players were more likely to be born in high-density communities (>1,000 residents/km^2^), yet handball players were more likely to be born in less-dense communities (100–250 residents/km^2^). Rossing et al. (13) continued this research by studying elite male (15–21 years old) Danish soccer players, again finding that that high-density communities (>1,000 residents/km^2^) produced more players. Similarly, van Nieuwstadt et al. (14) noted that increased urbanity (i.e., population density of one's community) elevates the likelihood of attaining professional status in Dutch male soccer, though the authors noted other variables (e.g., migration status and income) might influence the strength of urbanity effects. Switching sports, Hancock et al. (3) explored professional and semi-professional Portuguese volleyball players. For female players, being born in communities of differing population density did not influence their resulting competitive levels. For male players, however, being born in communities with lower population density (~300 residents/km^2^) increased the likelihood of achieving the highest competitive divisions compared to athletes born in communities with higher population density (~400 residents/km^2^).

Though population density might better reflect community structures, it still seems that results differ based on sport and country. As such, researchers have continued to employ other metrics to explore birthplace effects. These include considering proximity to the nearest soccer clubs (12), the concentration of basketball clubs in a region (11), and distance to larger cities that house developmental ice hockey teams (15). These and previously discussed metrics all yield the same conclusions: it evident that one's birthplace influences talent development in sport—even if the key demographic is different across sports and countries. Thus, it is imperative to understand the mechanisms that underpin the effect.

Hancock et al. (3), and later on Hancock (16), offered two mechanisms to explain birthplace effects. First, *infrastructure* captured environmental structures available to youth athletes that might facilitate talent development. Examples include training facilities, competition venues, available parks and green spaces for unstructured play, and teams or clubs that focus on development rather than winning. Of note, training and competition venues need not be state-of-the-art (often the case in large cities); rather, the venues merely need to be nearby and available for frequent use. Second, *social structure* centers on family and community factors that yield positive sport outcomes. The authors posited that social structure includes autonomy-supportive coaches, involved (but not over-involved) and caring parents, environments where unsupervised youth feel safe, and sport programs that promote positive youth development. The notion that optimal social structures yield favorable birthplace effects was previously intimated by Hancock and Côté (17), who stated that social agents (i.e., parents, coaches, and athletes) significantly influenced birthplace effects through mechanisms related to the self-fulfilling prophecy. Ultimately, communities that offer beneficial infrastructures and social structures are believed to produce more athletes, both at the elite and recreational levels. The proposed explanations—which are guided by logic and critical analysis of literature in similar research fields—provide some insights into birthplace effects. Nevertheless, they lack direct empirical support. To overcome our limited understanding of birthplace effects, it is vital to address the methodologies used in this research field.

## Weaknesses and gaps in birthplace methods

In any research venture, robust and varied methods are required to elucidate reliable and meaningful results that lead to strong conclusions regarding the data. The field of birthplace effects, however, suffers from many weaknesses and gaps that render it difficult to extract precise meaning from the research. Several factors contribute to this issue, which are explained herein.

The first such factor is the term “birthplace” itself. Likely due to ease of analysis, birthplace is the accepted proxy from which researchers draw conclusions regarding talent development. This presents several concerns, though developing superior and feasible alternatives has yet to happen. In many sports, it takes 10 or more years of athletic development to attain elite status. For some athletes, their birthplace and their community during the formative developmental years are one and the same. Many other athletes, however, are born in one community and then move to a different community before the age of entry into sport. Similarly, it is not uncommon to hear of athletes who begin their sport careers while living in one community, but then move to a different community after one, five, or even 10 years of development. For all these reasons, the nature of birthplace effects research has inherent limitations. Compounding this, birthplace is typically self-reported. In writing this chapter, the first author asked his son, “What do you consider to be your birthplace?” His son stated one city (population ~1,000,000), though we moved to a different city (population ~125,000) before he was 6-weeks-old. If asked again in 10 years, he might state the latter city as his birthplace, likening it more to a “home town.” Since the populations of these two cities are quite different, his response to that question could lead to different interpretations—similar to what one would expect of athlete populations when asked the same question. Not only is birthplace self-reported, but in most instances, data are collected by sport organizations, not researchers. Since there would rarely be a need for sport organizations to differentiate between birthplace and community of development, it likely means that little guidance is given to athletes about what constitutes birthplace. The nature of birthplace as defined and measured in the literature has several limitations that contribute to our lack of understanding of birthplace effects. Researchers would be wise to acknowledge these issues, while also seeking data collection techniques that rely on direct information from participants rather than archival methods.

A second concern relates to the community sizes that constitute each population category in birthplace effects research. Across the globe, many municipal governments have amalgamated to form larger cities with fewer administrative costs and streamlined—or at least centralized—community services. This raises a concern for birthplace effects researchers, who must deliberate on the population categorizations of amalgamated cities. Going back to the first author's son, his actual birthplace was a suburb of the larger city, which had a population of ~25,000. This brings into question what his assigned birthplace population size should be: 25,000 (the size of the suburb) or 1,000,000 (the size of the amalgamated city)? Typically, more accurate results come from specifying suburbs and amalgamated regions, though when reliant on sport organizations to provide birthplace data, researchers must accept whatever data were collected, even if they are not ideal. Again, this points to the need for researchers in this field to consider direct data collection measures, along with clear decision processes for categorizing various community sizes, to improve the validity of their findings.

Recent research overseen by Wattie (15, 18, 19) highlight a third issue with birthplace effects research: assuming cities of equal size and density are homogeneous. Wattie et al. (18) identified that, in 1996, 36% of residents in the Canadian province of Ontario lived in cities of greater than 250,000 people. Meanwhile, in Canada's four Atlantic provinces at that time, no cities existed of that size. In essence, what is considered a medium city in one region could be viewed as a large city in another. Extending this principle, small communities should not be deemed homogeneous simply because they exist in the same country. To illustrate this point, consider the cities of Shelburne, Ontario (~8,000 residents) and Estevan, Saskatchewan (~13,000 residents). Shelburne is certainly a small community, but residents are a two-hour drive or less (assuming little traffic) to nine other centers with 100,000 or more residents. Meanwhile, Estevan is also a small community, but is slightly more than a two-hour drive to the nearest center with 100,000 or more residents. As Wattie et al. (18) and Farah et al. (19) rightly indicated, it is because of differences such as these that communities of equal size should not be treated homogeneously. Instead, researchers must endeavor to explore the underlying infrastructures and social structures that drive birthplace effects, grouping communities together for analysis only when their structures are truly homogeneous.

A final weakness related to birthplace effects is the type of research that is typically conducted. Most often, researchers employ archival methods (e.g., collecting birthplaces from websites) to explore birthplace effects. Such approaches have been vital in identifying the presence of birthplace effects across sports and countries, but they have been less useful for contributing to our understanding of why birthplace effects exist. Instead, researchers ought to seek varied methods for future research including direct participant interactions (as noted above), longitudinal or quasi-longitudinal designs (to track changes over time and shifts in the communities in which athletes live), and qualitative methods (learning about participants' experiences in small, medium, and large communities).

The fact that this section is longer than the preceding ones speaks volumes to how limited our knowledge is of birthplace effects. This is partially because it is a field still in its infancy, but also because several weaknesses in researchers' approaches render it challenging to draw firm conclusions about the field. Hopefully those reading this chapter (students or researchers) take it as a call to action and are inspired to create research designs with the goal of standardizing birthplace effects measures/metrics that lead to meaningful conclusions.

## Talent hotspots in North American basketball

The following section aims to draw further attention to some of the gaps in the birthplace effect literature identified above. In response to critiques challenging the homogeneity of communities of particular sizes (18, 19), we present data from a study which investigated potential birthplace effects in men's and women's basketball in the United States at both the collegiate and professional levels.^1^ Through the examination of birthplace effects in terms of both absolute population and population density, we aim to identify potential “talent hotspots” that can be used to further advance discussions of the underlying mechanisms driving birthplace effects. In addition, the investigation of birthplace effects in a single sport in both male and female and collegiate and professional levels of competition will help to better understand how the birthplace effect manifests across sport contexts.

### Participants

A total of 8,740 American professional and collegiate basketball players were included in the study. Places of birth were collected from the official websites of the National Basketball Association (NBA; *n* = 382), Women's National Basketball Association (WNBA; *n* = 120), Men's Division I National Collegiate Athletic Association (MNCAA; *n* = 4,030), and Women's Division I National Collegiate Athletic Association (WNCAA; *n* = 4,208) using each team's 2018 rosters.

### Procedure

Birthplace data were collected for each of the four leagues, and individuals born outside of the United States were excluded from the analyses. United States census statistics were then used to compare athletes' birthplace information with the general population for both community size and density. To account for the average age differential between the professional and collegiate samples, two separate sets of census data were collected. The 2000 United States census (20) was used for the NBA and WNBA datasets, while the 2010 United States census (21) was used for both the MNCAA and WNCAA.

### Data analysis

Communities were categorized into eight groups based on population size: (1) <50,000 residents, (2) 50,000–99,999 residents, (3) 100,000–249,999 residents, (4) 250,000–499,999 residents, (5) 500,000–999,999 residents, (6) 1,000,000–2,499,999 residents, (7) 2,500,000–4,999,999 residents, and (8) ≥5,000,000 residents. For population density, eight groups were used: (1) <50 residents/km^2^, (2) 50–99 residents/km^2^, (3) 100–249 residents/km^2^, (4) 250–499 residents/km^2^, (5) 500–999 residents/km^2^, (6) 1,000–2,499 residents/km^2^, (7) 2,500- 4,999 residents/km^2^, and (8) ≥5,000 residents/km^2^.

Odds ratios (ORs) were calculated across the different population sizes and densities for each group. ORs and 95% confidence intervals (CIs) were calculated by dividing the chance of being a part of the sample group from a population size or density (e.g., an NBA athlete) by the chance of being from a specific community with that population size or density based on the general population (i.e., Census data). ORs were interpreted based on their positioning above or below 1. An OR above 1 implies that an athlete in that population size or density has a greater likelihood of participating at elite levels compared to an individual from a different population size or density. Conversely, ORs less than 1 means that an athlete is less likely to achieve success from a certain population size or density than someone from another area. If the CI contains the 1, those ORs are deemed not statistically significant.

## Results

### Collegiate athletes

There was a significant over-representation of both male and female NCAA athletes from two community size categories, ranging from 100,000 to 499,999 residents (Table 1). This effect was the strongest for athletes born in cities of 250,000–499,999, with 11.05% of male NCAA athletes and 10.31% of female NCAA athletes compared to just 6.03% of the general US population (OR_MNCAA_ = 1.94, CI_MNCAA_ = 1.75–2.14; OR_WNCAA_ = 1.79, CI_WNCAA_ = 1.62–1.98). While the specific ORs differed slightly across male and female NCAA athletes, the overall trends in relation to community size were consistent across genders.

**Table 1:** Odds ratios (ORs) and confidence intervals (CIs) across city size categories for collegiate athletes.

**Community size**	**US (%)**	**MNCAA (%)**	**OR**	**CI**	**WNCAA (%)**	**OR**	**CI**
≥5,000,000	2.58	0.25	0.09	0.05–0.18	1.00	0.38	0.28–0.52
2,500,000–4,999999	4.47	4.15	0.92	0.79–1.08	2.28	0.50	0.41–0.61
1,000,000–2,499,999	7.50	6.63	0.88	0.77–0.99	4.52	0.58	0.50–0.67
500,000–999,999	7.73	10.75	1.44	1.30–1.59	7.73	1.03	0.92–1.16
250,000–499,999	6.03	11.05	1.94	1.75–2.14	10.31	1.79	1.62–1.98
100,000–249,999	11.82	15.35	1.35	1.24–1.47	15.02	1.32	1.21–1.44
50,000–99,999	13.13	12.91	0.98	0.89–1.08	13.36	1.02	0.93–1.11
<50,000	46.74	38.91	0.73	0.68–0.77	44.75	0.92	0.87–0.98

As for population density, there was a significant over-representation of male and female collegiate athletes from moderately dense cities between 500 and 2,499 residents/km^2^ (Table 2), with the largest ORs observed for the 1,000–2,499 residents/km^2^ category (OR_MNCAA_ = 1.38, CI_MNCAA_ = 1.30–1.47; OR_WNCAA_ = 1.36, CI_WNCAA_ = 1.23–1.40). Similar to the community size data, ORs followed similar trends across density categories in both male and female NCAA athletes.

**Table 2:** Odds ratios (ORs) and confidence intervals (CIs) across population density categories for collegiate athletes.

**Population density (inhabitants/km^2^)**	**US (%)**	**MNCAA (%)**	**OR**	**CI**	**WNCAA (%)**	**OR**	**CI**
≥5,000	14.96	11.82	0.76	0.69–0.84	8.51	0.53	0.47–0.59
2,500–4,999	13.70	14.65	1.08	0.99–1.18	14.52	1.07	0.98–1.17
1,000–2,499	40.19	48.17	1.38	1.30–1.47	47.74	1.36	1.23–1.40
500–999	17.11	18.6	1.11	1.02–1.12	20.46	1.25	1.16–1.34
250–499	6.54	4.92	0.74	0.64–0.85	4.78	0.72	0.62–0.83
100–249	3.74	1.24	0.32	0.26–0.43	1.78	0.47	0.37–0.59
50–99	1.75	0.25	0.14	0.08–0.26	0.40	0.23	0.14–0.37
<50	2.05	0.35	0.17	0.01–0.28	0.33	0.16	0.10–0.27

### Professional athletes

Among both NBA and WNBA datasets, there was a significant over-representation of male and female participants from three community size categories ranging from 100,000 to 999,999 residents (see Table 3). For the NBA, this relationship was the strongest for athletes from cities ranging from 250,000 to 499,999 residents (OR_NBA_ = 2.90, CI_NBA_ = 2.20–3.81), while the WNBA showed the largest over-representation in the 500,000–999,999 residents category (OR_WNBA_ = 3.12, CI_WNBA_ = 1.96–4.95). Besides the ranges noted above, NBA athletes were also significantly over-represented from cities between 50,000 and 99,999 residents (OR_NBA_ = 1.33, CI_NBA_ = 1.01–1.76) and 2,500,000–4,999,999 residents (OR_NBA_ = 2.41, CI_NBA_ = 1.65–3.52).

**Table 3:** Odds ratios (ORs) and confidence intervals (CIs) across city size categories for professional athletes.

**Community size**	**US (%)**	**NBA (%)**	**OR**	**95% CI**	**WNBA (%)**	**OR**	**95% CI**
≥5,000,000	2.78	0.52	0.18	0.05–0.73	3.33	1.21	0.45–3.27
2,500,000–4,999999	3.29	7.59	2.41	1.65–3.52	3.33	1.01	0.37–2.74
1,000,000–2,499,999	9.6	11.78	1.26	0.92–1.71	6.67	0.67	0.33–1.38
500,000–999,999	6.75	12.83	2.04	1.5–2.76	18.33	3.12	1.96–4.95
250,000–499,999	6.16	15.97	2.90	2.20–3.81	11.67	2.01	1.15–3.52
100,000–249,999	11.14	15.97	1.52	1.15–1.99	23.33	2.43	1.59–3.71
50,000–99,999	12.26	15.71	1.33	1.01–1.76	9.17	0.72	0.39–1.34
<50,000	48.06	23.04	0.32	0.26–0.41	24.2	0.34	0.23–0.52

As for population density, there was an over-representation for both the NBA and WNBA in relatively dense cities (Table 4). For the WNBA, extremely dense cities, (≥5,000 residents/km^2^) were 8.3 times more likely to produce professional basketball players (OR_WNBA_ = 8.30, CI_WNBA_ = 5.77–11.94). For the NBA, the only population density category that was significantly over-represented was 2,500–4,999 residents/km^2^ (OR_NBA_ = 1.71, CI_NBA_ = 1.35–2.17), which was also over-represented in the WNBA sample (OR_WNBA_ = 1.85, CI_WNBA_ = 1.21–2.81).

**Table 4:** Odds ratios (ORs) and confidence intervals (CIs) across population density categories for professional athletes.

**Population density (inhabitants/km^2^)**	**US (%)**	**NBA (%)**	**OR**	**95% CI**	**WNBA (%)**	**OR**	**95% CI**
≥5,000	14.87	16.23	1.11	0.85–1.46	59.17	8.30	5.77–11.94
2,500–4,999	14.71	22.77	1.71	1.35–2.17	24.17	1.85	1.21–2.81
1,000–2,499	39.53	43.72	1.12	0.97–1.45	14.17	0.25	0.15–0.42
500–999	16.76	12.83	0.73	0.54–0.99	0.83	0.04	0.01–0.30
250–499	6.38	0.52	0.08	0.02–0.31	0.83	0.12	0.02–0.83
100–249	3.6	0.52	0.14	0.04–0.57	0.83	0.23	0.03–1.61
50–99	1.88	0	0	–	0	0	–
<50	2.27	0	0	–	0	0	–

## Discussion

The purpose of this study was to analyze the birthplace effects in both collegiate and professional United States basketball based on population size and density. By collecting data from two competitive levels and sexes, we were able to extend upon extant literature by providing a more nuanced understanding of birthplace effects in the sport of basketball in the United States. Notably, the largest over-representation in community size for NBA athletes were from cities between 250,000 and 499,999 residents, while WNBA athletes were most over-represented from cities between 500,000 and 999,999 residents. For both collegiate males and females, the highest representation was from cities between 250,000 and 499,999 residents. For population density, NBA athletes were most likely to be from moderately dense areas (2,500–4,999 residents/km^2^), while WNBA athletes were highly over-represented in extremely dense population centers (≥5,000 residents/km^2^).

Referring to population size, both collegiate and professional datasets indicate that medium- to large-sized cities might be the most conducive to reaching elite-level performance, which aligns with previous findings in other professional and Olympic sports (6). However, these findings also suggest that since Côté et al.'s (1) initial study of the birthplace effect in the NBA, there appears to be a shift in optimal city size from small and medium cities (100,000–249,999 residents) to medium and large cities (250,000–999,999 residents). Given that researchers often credit smaller communities for fostering personal and talent development (4), it is possible that this shift to larger cities is due to other factors such as better competition and more opportunities. For example, the popularity of the Amateur Athletic Union (AAU) and other “elite” youth basketball clubs has drastically increased since 2010, with the majority of NBA athletes previously participating in the AAU, where tournaments are often nested in larger cities (22). Thus, this shift might be representative of increased opportunities to train and compete with highly skilled opponents, while importantly providing youth athletes with exposure to college scouts.

Additionally, the present study found that relatively dense populations were more conducive for both NBA and WNBA athletes (Table 4; 2,500–4,999 residents/km^2^). Similar findings were observed for collegiate athletes, who additionally were found to be heavily over-represented from medium density communities (Table 2; 1,000–2,499 residents/km^2^). Altogether, these findings were consistent with prior birthplace effect studies that have considered density in European populations (13). It is possible that these high-density centers, regardless of population size, are able to provide greater access to facilities, opportunities, and exposure to important sport cultural norms (12).

The present study has highlighted the shift from small to medium cities for professional basketball players with relatively high densities. It also provided additional analyses necessary in elite level basketball by including NCAA Division I athletes. However, this study has several limitations and considerations for future research. The included sample contained male and female basketball players from the collegiate and professional ranks, but it is not clear as to where the most elite players are coming from. For example, while the NCAA includes over 350 teams in Division I basketball, the majority of future professionals are recruited from a much smaller group of highly touted, well-resourced programs, while many smaller Division I institutions produce very few (if any) professionals (23).

One of the unique objectives of this study was to identify potential “talent hotspots” in both men's and women's basketball in the US. Through the analysis of the optimal community sizes and densities at both the collegiate (where most professional players are drafted from) and professional ranks, it was possible to identify community sizes and densities that are over-represented at both levels of competition. In men's basketball, there was a significant over-representation of collegiate and professional players from communities with populations of 100,000–999,999 residents. In terms of population density, there were no significant overlapping ORs across the men's collegiate and professional datasets. However, both data sets favor relatively dense communities (i.e., 500–4,999 residents/km^2^). In women's basketball, there was a significant over-representation of both collegiate and professional players from communities with populations of 100,000–499,999, with the data skewing toward increasingly dense communities in the transition from the collegiate (i.e., 500–2,499 residents/km^2^) to professional ranks (i.e., 2,499–≥5,000 residents/km^2^). Finally, in examination of collegiate and professional men's and women's basketball altogether, it is evident that communities of 100,000–249,999 residents can be considered “talent hotspots” as there are significant over-representations of athletes from communities of this size across all four data sets.

Through the identification of these potential “talent hotspots” in terms of both community size and density in elite basketball in the United States, we have presented a worthwhile avenue for future research. While these data were cross-sectional in nature, the identification of multiple consistent “talent hotspots” along the pathway to professional basketball helps to better understand the developmental nature of birthplace effects. Follow-up studies may wish to pinpoint specific communities that meet these population size and density criteria and investigate those which are most frequently observed in the sample of NBA and WNBA players. In doing so, it will be possible to go beyond just considering how infrastructure (related to community size) and social structure (related to density) impact athlete development, to begin exploring other indices of these communities that may be optimal for the development of elite basketball players [e.g., green space, organizations, and social norms (3)].

## Key takeaway messages for the geography of talent development

It is evident that geography is a significant factor influencing talent development among athletes. Likely, this is because of favorable infrastructure and social structure that exist in certain communities. As indicated by the data presented herein, communities with optimal population sizes and densities might be considered talent hotspots. However, researchers ought to continue exploring other elements within such communities (e.g., crime rates and green spaces) to deepen our understanding of these potential hotspots. Such explorations might also identify environmental/geographic factors that consistently explain talent development advantages across sports and countries.

## Data availability statement

The raw data supporting the conclusions of this article will be made available by the authors, without undue reservation.

## Author contributions

DH led the writing of most sections of the paper. MV led the writing of the final section which includes the original research. AN contributed with article reviews, referencing, and reviewing. All authors contributed to the article and approved the submitted version.

## Conflict of interest

The authors declare that the research was conducted in the absence of any commercial or financial relationships that could be construed as a potential conflict of interest.

## Publisher's note

All claims expressed in this article are solely those of the authors and do not necessarily represent those of their affiliated organizations, or those of the publisher, the editors and the reviewers. Any product that may be evaluated in this article, or claim that may be made by its manufacturer, is not guaranteed or endorsed by the publisher.

## Footnotes

^1^In the United States, professional basketball players are drafted from the collegiate ranks.
